# Extensive Ureteral Stricture in Renal Transplant Recipients: Prevalence and Impact on Graft and Patient Survival

**Published:** 2013-11-01

**Authors:** R. Mahdavi Zafarghandi, Zh. Sheikhi

**Affiliations:** 1 *Department of Urology and Renal Transplantation, Emam Reza Hospital, Mashhad University of Medical Sciences, Mashhad, Iran *; 2 *School of Medicine, North Khorasan University of Medical Sciences, Bojnurd, Iran*

**Keywords:** Ureter, Renal transplant, Prevalence, Survival

## Abstract

Extensive ureteral stricture (EUS) after renal transplantation (RTx) is an important urological complication that adversely affects the longterm function of the allograft and therefore the morbidity and mortality of the recipients. We conducted this study to determine the prevalence of the EUS in RTx recipients and its impact on the patient and graft survival. We assessed retrospectively, 1450 patients who underwent renal transplantation by a fixed surgical team between December 1991 and December 2009 at Emam Reza Hospital, Mashhad University of Medical Science, Mashhad, Iran. EUS was diagnosed in 13 (1.1%) patients including 8 (61.5%) male. The mean±SD age of patients at the time of surgery was 33.6±13.7 years; the length of follow-up was 77.9±63.5 months; and the ischemic time was 126.5±114.1 min. Mostly, EUS was noticed in recipients of transplants with more than one artery (p<0.05) and of cadaveric donors with more than 4 hour ischemic time (p<0.001). In follow-up, after ureteropyelostomy (7 cases), ipsilateral pyelopyloplasty (4 cases) and contralateral pyelopyeloplasty (2 cases), no evidence of ureteral stricture recurrence, graft loss or death was observed. We concluded that the incidence of EUS, as a urologic complication after RTx is very low. The advanced techniques of RTx that preserve the ureteric blood supply and the better procedures for ureteral reconstruction have improved the survival rate of patient and graft.

## INTRODUCTION

Renal transplantation (RTx) is the ultimate treatment for patients with end-stage renal disease [1]. Urological complications remain a major source of morbidity and mortality in RTx patients; however, the incidence of these complications has halved over the last 30 years [2]. They occur approximately in 10% of RTx [2, 3] and include urinary fistula (distal ureteral necrosis), ureteral stricture, renal calculi, symptomatic vesicoureteral reflux and lymphocele. Ureteral stricture is assumed as one of the most important complications after RTx as well any other urological and gynecological surgery [4, 5]. It is estimated that the incidence is 0.6%–10.5% [6-8]; with newer techniques, extensive ureteral stricture (EUS) after RTx is very rare [9]. However, EUS is considered a highly important complication as it can adversely affect the morbidity and mortality of the recipient [10]. 

The time of presentation and etiology of ureteral strictures are different. Early strictures are mainly due to the technical errors at retrieval or reimplantation or compromised ureteral blood supply during surgery. The precise cause of late ureteral strictures is not fully known. Impairment of tissue healing secondary to inflammation, infection, anti-proliferative and immunosuppressive therapies, fibrosis or progressive vascular disease and nutritional regimes, is considered the main reason [11-15]. Whether the presentation is early or late and the etiology is technical or inflammatory, the most considerable point is that ureteral strictures can lead to the failure of allograft and deteriorate the morbidity and mortality of the recipient [16]. Therefore, early diagnosis and correction of the strictures are of paramount importance [17, 18]. The treatment of choice includes an endourological or ureteroneocystostomy procedure [18-20]. Ureteropyelostomy, ipsilateral pyelopyloplasty and contralateral pyelopyeloplasty have been performed to correct the strictures [20]. This study was designed to determine the prevalence of EUS in RTx and the impact of EUS on the survival of the graft and recipient.

## MATERIAL AND METHODS

**Figure 1:**
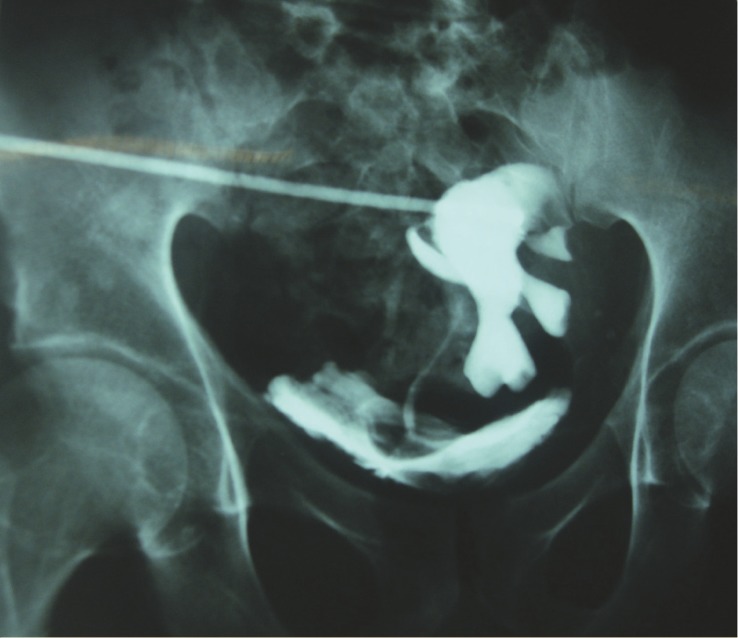
Antegrade pyelography in a patient with extensive ureteral stricture

In a retrospective study conducted between December 1991 and December 2009 at Emam Reza Hospital, Mashhad University of Medical Science (MUMS), Mashhad, Iran, 1450 patients who had underwent RTx by a fixed surgical team were evaluated to determine the prevalence of post-RTx EUS . The study was approved by the regional ethics committee of MUMS. Based on the results of antegrade pyelography and excretory urography (Fig 1), diagnosis of ureteral stricture was made in the patients presenting with increased serum creatinine levels and/or hydronephrosis of the graft on ultrasonography. The initial treatments of all the patients were insertion of percutaneous nephrostomy tube. All the patients received immunosuppression consisted of cyclosporine (600 mg/pkg/day), prednisolone (1mg/kg/day, after 3months taper to 15 mg/day), and mycofenalatmophenat (2 g/day)*.* The indication for surgical treatment (ureteral reconstruction or continuity of the upper urinary tract) included existence of EUS. In our patients, the mean length of EUS was 4.4 (range: 3.5–5) cm. 

**Figure 2:**
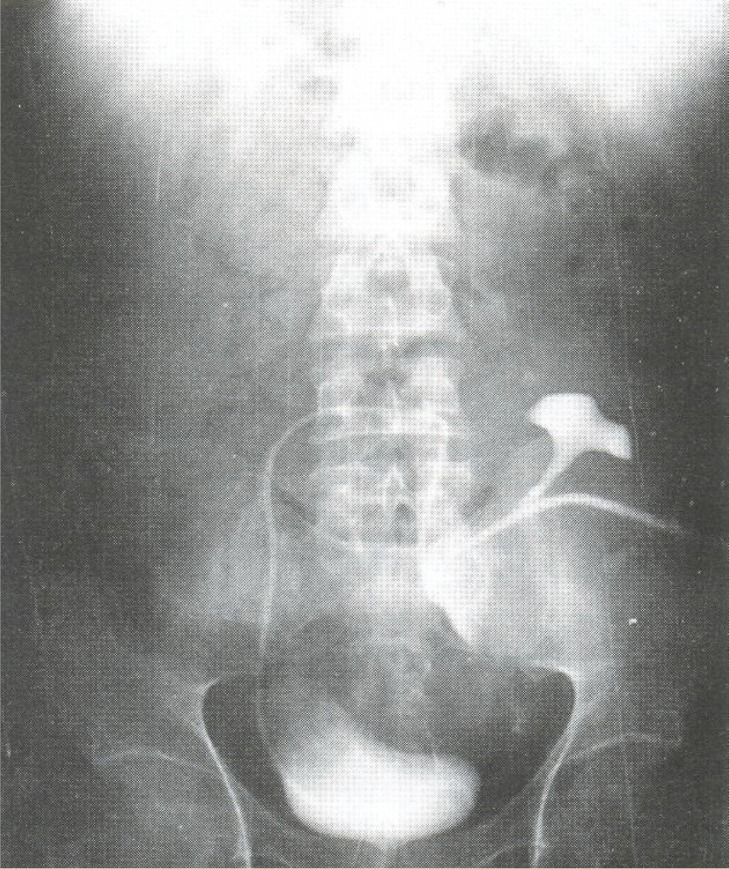
The course of the contralateral native ureter after pyelopyeloplasty

All patients with EUS underwent general anesthesia and reconstruction for continuity of the upper urinary tract. They had a nephrostomy tube during and at least four weeks after the procedure. The reconstruction techniques performed included uretero pyelopyeloplasty (surgical anastomosis between ipsilateral native ureter and pelvis of transplanted kidney); ipsilateral pyelopyeloplasty (surgical anastomosis between pelvis of ipsilateral native kidney and pelvis of transplanted kidney); and contralateral pyelopyeloplasty (anastomosis between pelvis of contralateral native kidney and pelvis of transplanted kidney. In this procedure, after nephrectomy of contralateral native kidney and discard atrophic renal paranchyma, its pelvis and ureter were transferred to other side via posterior peritoneum (Fig 2). After the surgical repair, a double J stent was inserted for all patients. 

## RESULTS

EUS was diagnosed in 13 (1.1%) patients including eight (61.5%) male. EUS was determined in seven patients at the first three months, two patients at the second three months, and in one after the seventh month post-transplantation. The mean±SD age of patients at the time of surgery was 33.6±13.7 (range: 10–60) years; the ischemic time was 126.5±114.1 (range: 25–300) minutes; and the length of follow-up was 77.9±63.5 (range: 12–228) months. In seven cases (54%), the source of the allograft was non-relative living donor; five (39%) had cadaveric donors; and only one patient (8%) had a relative living donor.

There was no difference between males and females in terms of occurrence of EUS. No significant correlation was observed between the age of patients and development of EUS. Mostly, EUS was noticed in recipients of transplants with more than one artery (p<0.05) and of cadaveric donors with more than four hour cold ischemic time (p<0.001). There were no association between gender and ischemic time; source of the allograft and ischemic time; size of stricture and source of the allograft; and ischemic time and age.

The surgical procedure for correction was ureteropyelostomy (7 cases), ipsilateral pyelopyloplasty (4 cases) and contralateral pyelopyeloplasty (2 cases) (Table 1). We followed patients post-operatively with ultrasound every three months until the end of the first year, then every 6 months in the second year, and annually, thereafter. The patients were also followed by other imaging modalities such as MRI and IVP, if necessary. There was no restenosis. During the follow-up no graft loss or death was recorded. EUS had no effect on 3-, 5- and 10-year graft and patient survival, after the surgical correction.

**Table 1:** The characteristics of patients with extensive ureteral stricture after renal transplantation

Patient number	Year of transplant	Age at surgery (years)	Follow-up length (months)	Source of donor	Sex	Ischemic time (min)	Stricture length (cm)	No. of arteries in the graft	Procedure	Graft loss	UTI	death
1	1991	35	228	Non-relative	Female	35	3.5	1	Ureteropyeloplasty	-	-	-
2	1997	22	157	Non-relative	Male	45	2.5	2	Ureteropyeloplasty	-	-	-
3	2000	10	126	Relative	Male	45	8.0	2	Contralateral pyelopyeloplasty	-	-	-
4	2002	30	102	Non-relative	Female	25	4.0	1	ipsilateral pyelopyeloplasty	-	-	-
5	2003	45	91	Non-relative	Male	40	3.0	1	Ureteropyeloplasty	-	-	-
6	2003	22	86	Cadaveric	Male	240	4.0	1	ipsilateral pyelopyeloplasty	-	-	-
7	2005	51	63	Cadaveric	Male	270	3.0	1	Ureteropyeloplasty	-	-	-
8	2007	22	40	Non-relative	Female	45	8.0	2	Contralateral Ureteropyeloplasty	-	-	-
9	2007	18	36	Cadaveric	Male	240	6.0	1	Ureteropyeloplasty	-	-	-
10	2008	35	31	Cadaveric	Male	300	7.0	1	ipsilateral pyelopyeloplasty	-	-	-
11	2008	60	22	Non-relative	Male	5	6.0	1	Ureteropyeloplasty	-	-	-
12	2009	40	19	Cadaveric	Female	270	5.0	1	ipsilateral pyelopyeloplasty	-	-	-
13	2009	38	12	Non-relative	Female	40	6.0	2	Ureteropyeloplasty	-	-	-

## DISCUSSION

Having a prevalence of nearly 1%, we found that EUS is no longer a common complication after RTx. This observation was in keeping with other reports. Safa, *et al*, reported that the incidence of distal ureteral stricture was 5.6%; there was no EUS in their series [14]. Shoskes, *et al*, reported ureteral stricture after RTx in 3.6% of patients; there was also no EUS [11]. EUS is considered a late complication of RTx. Therefore, longer follow-up seems necessary to find EUS.

Neither age nor gender was important in the development of EUS; however, we found that there was a significant association between the duration of cold ischemia and development of EUS. This certainly supported the reports suggesting the role of ischemia in development of EUS [14]. Other studies also supported this hypothesis [21, 22]. We found that increased ischemic time and number of renal arteries involved contribute to the development of post-RTx EUS. However, inflammation, infection, immunosuppressive and anti-proliferative therapy might be other possible responsible factors [14]. Another important determinant is the surgical technique used for graft retrieval and reimplantation, as it can significantly reduce the incidence of complications [16, 23-25]. For instance, it is documented that in children, ureteroureteral anastomosis is a safe and effective technique with low complication rates. The authors explained that a better vascularization of the shorter ureteral end of the graft might be the reason for lower incidence of EUS [23]. Another study showed that Taguchi ureteroneocystostomy had higher complication rates compared to Lich-Gregoir technique [25].

The surgical treatment of EUS depends on several parameters such as length of the stricture, degree of the fibrosis, *etc*. The most important point is that evaluation and surgery must be done as soon as possible in order to save precious time and preserve the function of kidney. We performed ureteropyeloplasty, ipsilateral pyelopyloplasty and contra-lateral pyelopyeloplasty (Fig 2), for ureteral reconstruction. Contralateral ureteropyeloplasty was performed for patients with a non-suitable Boari flap for total replacement of the ureter. We substituted the pelvic and ureter of nephrectomized contralateral native kidney. If the stricture is very long to be reimplanted, artificial ureter or intestinal segments must be used. Contralateral ureteropyeloplasty technique is cheaper and has less complications than other techniques [26]. All of these surgical procedures were successful, and none of them was associated with a poor outcome or death. 

It should be noticed that there are different opinions regarding the treatment of EUS after RTx. While many surgical techniques have been introduced, it seems that a minimal invasive technique like endourological procedure may be useful in some patients [22]; nonetheless, we found that open surgery and open plastic surgery is the final procedure for salvaging the transplanted kidney. 

One of the limitation of our study was that we only surveyed data from EUS patients; we did not survey all RTx patients’ data and thus we could not compare the EUS patients with all patients’ data.
